# The Robust Running Ape: Unraveling the Deep Underpinnings of Coordinated Human Running Proficiency

**DOI:** 10.3389/fpsyg.2017.00892

**Published:** 2017-06-12

**Authors:** John Kiely

**Affiliations:** Institute of Coaching and Performance, School of Sport and Wellbeing, University of Central LancashirePreston, United Kingdom

**Keywords:** running, rehabilitation, coordination, overuse, variability, plasticity

## Abstract

In comparison to other mammals, humans are not especially strong, swift or supple. Nevertheless, despite these apparent physical limitations, we are among Natures most superbly well-adapted endurance runners. Paradoxically, however, notwithstanding this evolutionary-bestowed proficiency, running-related injuries, and Overuse syndromes in particular, are widely pervasive. The term ‘coordination’ is similarly ubiquitous within contemporary coaching, conditioning, and rehabilitation cultures. Various theoretical models of coordination exist within the academic literature. However, the specific neural and biological underpinnings of ‘*running coordination,’* and the nature of their integration, remain poorly elaborated. Conventionally running is considered a mundane, readily mastered coordination skill. This illusion of coordinative simplicity, however, is founded upon a platform of immense neural and biological complexities. This extensive complexity presents extreme organizational difficulties yet, simultaneously, provides a multiplicity of viable pathways through which the computational and mechanical burden of running can be proficiently dispersed amongst expanded networks of conditioned neural and peripheral tissue collaborators. Learning to adequately harness this available complexity, however, is a painstakingly slowly emerging, practice-driven process, greatly facilitated by innate evolutionary organizing principles serving to constrain otherwise overwhelming complexity to manageable proportions. As we accumulate running experiences persistent plastic remodeling customizes networked neural connectivity and biological tissue properties to best fit our unique neural and architectural idiosyncrasies, and personal histories: thus neural and peripheral tissue plasticity embeds coordination habits. When, however, coordinative processes are compromised—under the integrated influence of fatigue and/or accumulative cycles of injury, overuse, misuse, and disuse—this spectrum of available ‘choice’ dysfunctionally contracts, and our capacity to safely disperse the mechanical ‘stress’ of running progressively diminishes. Now the running work burden falls increasingly on reduced populations of collaborating components. Accordingly our capacity to effectively manage, dissipate and accommodate running-imposed stress diminishes, and vulnerability to Overuse syndromes escalates. Awareness of the deep underpinnings of running coordination enhances conceptual clarity, thereby informing training and rehabilitation insights designed to offset the legacy of excessive or progressively accumulating exposure to running-imposed mechanical stress.

## Introduction

Running is the most primitively ancient of athletic movements: critical to competitive success in many sports and, in evolutionary contexts, critical to survival. Uniquely amongst mammals humans employ an upright bipedal bouncing gait when running. A gait characterized by long flight times interspersed with brief ground contacts during which the shock of impact, equating to multiple times bodyweight, is absorbed, re-cycled, and steered through the narrow stabilizing platform provided by a single supporting foot. Nevertheless, despite these apparent limitations, we are amongst Nature’s most supremely well-adapted runners (Bramble and Lieberman, 2004).

The evolutionary innovations bestowing human running proficiency do not, however, render us invulnerable to breakdown and running-related injuries are common (van der Worp et al., 2015). Runners seem particularly exposed to Overuse injuries, with up to 70% suffering such injury each year (Clarsen et al., 2013; Saragiotto et al., 2014; van der Worp et al., 2015). Various definitions exist, amid some inconsistency, and confusingly ‘Overuse’ describes both a ‘mechanism’ and ‘type’ of injury (Clarsen et al., 2013). Although definitions vary, published consensus agrees that Overuse syndromes arise consequent to progressively mounting micro-trauma accumulated over a protracted period, exacerbated by insufficient recovery leading to increasing tissue sensitization in the absence of single catastrophic events (Clarsen et al., 2013; Saragiotto et al., 2014). Commonly cited risk factors include elevated running volumes, prior injury, fatigue and background psychosocial stress (Clarsen et al., 2013; van der Worp et al., 2015; Ivarsson et al., 2016). Yet how these factors synergistically interact, leading to Overuse injuries, has yet to be clarified (van der Worp et al., 2015).

A frequently overlooked distinction between running and many other sporting movements is that running is one of a limited sub-set of gaits—along with crawling and walking—that are so evolutionary ancient as to have mutually co-evolved in tandem with human neural and biological infrastructures (Kiely and Collins, 2016). In short: how we run is shaped by, yet has also contributed to shaping, modern human morphology, in ways that other sporting movements—a golf swing; a tennis serve; rowing; the butterfly stroke—, have not. An implication of this synergistic co-evolution of form and function is that the adaptations underpinning human running permeate every dimension of our anatomical, biological, and neurological being. Our capacity to withstand the extraordinary mechanical and stability challenges imposed during our bouncing bipedal running gait is not attributable to any single evolutionary adaptation. Instead human running robustness emerges as a consequence of our slowly developing capacity to seamlessly harness, orchestrate and integrate the outputs of multiple biological and neurological sub-systems to accomplish running objectives. In short: our ability to coordinate the running action.

The core defining feature of coordination is that multiple components work together to realize an objective (Diedrichsen et al., 2010). Conventionally, within the Sports Sciences, coordination is perceived through the lens of Dynamical Systems Theory (DST). Recently, through the lens of Optimal Feedback Control Theory (OFCT), conventional interpretations of DST have been criticized for obscuring the fundamental priority of sensory feedback in shaping effective movement coordination (Todorov, 2004, 2009). The OFCT framework subsequently claims to more prominently highlight the relationship between high-level goals, and the real-time sensorimotor control strategies most suitable for accomplishing those goals. Recent ecological dynamics perspectives have similarly advocated the prominent role of emerging sensory ‘information’ in regulating on-going motor behavior (Seifert et al., 2013). As in other scientific domains, however, debates and disagreements exist and the need for on-going argument, skepticism and scrutiny remain obvious. Various perspectives, accordingly, have been expertly and extensively discussed within their respective motor control and neuroscientific literatures (see for example: Davids and Glazier, 2010; Nagengast et al., 2010; Kelso, 2012; Proske and Gandevia, 2012). The problem, for the vast majority of practical Sports Scientists, Sports medicine practitioners and evidence-led Coaches is that while these academic debates are essential, by necessity they are abstract, highly technical, typically obscured by the in-house terminology of the specific academic realm and often too narrowly focused to provide practically implementable insight. Accordingly, any attempt to construct a coherent overview of such a diverse and contentious topic will, inevitably, be flawed and incomplete. Nevertheless, the overarching objective of this review is to provide this targeted group with an updated evidence-led synopsis of the key linked dimensions of the running coordination phenomenon deemed most relevant to performance, resilience and injury rehabilitation.

## The Evolutionary Undercurrents of Coordinated Running Robustness

Evolutionary survival demands that biological systems, operating in unpredictable environments using unreliable components and finite energy sources, are robust to the challenges to which they are most commonly exposed (Kitano, 2004). Accordingly, from an evolutionary perspective, running coordination’s overriding imperative is to deploy available resources to satisfactorily achieve desired outcomes, while preserving an acceptable robustness to any running-imposed ‘threat’ serving to reduce survival probability.

This ‘threat’ takes many forms. If energy depletes; if mechanical tissue tolerances are exceeded; if neural processes are overloaded to the extent that movement precision and/or cognitive clarity declines, then inevitably survival probability diminishes. No single survival imperative necessarily predominates. Instead the neurobiological system seeks to satisfactorily resolve multiple partially overlapping, partially competing organizational constraints (Skoyles, 2008; Hodges and Tucker, 2011; Miller et al., 2012). In negotiating this complex organizational problem, evolution has arrived at a typically ingenious resource-sparing set of solutions.

### Interpretation of Sensation Shapes Movement

As running increases in severity we are made consciously aware of mounting ‘threat’ through increasingly discomforting interpretations of arising sensory information (Marcora et al., 2009; Smirmaul, 2012). At the whole-body level growing discomfort influences psycho-emotional state, amplifying perceptions of anxiety, ‘pain’ and diminished attention which in turn intensify the inner conflict between motivational drive and perceived effort that, collectively, we interpret as mounting ‘fatigue’ (Marcora et al., 2009; Seay et al., 2011; Smirmaul, 2012). At the local level, muscle activation patterns are subtly modulated to offload sensitized tissues, thereby moderating regionalized discomfort and alleviating tissue irritation (Mizrahi et al., 2000; Gerlach et al., 2005; Seay et al., 2011). Through these mechanisms our interpretation of arising psychobiological discomfort informs us of increasing risk—of impending tissue damage, elevating metabolic costs, increasing neural processing demands and cognitive effort—, thereby providing a direct means through which the perceived relevance of changing sensation directly influences running behavior (Marcora et al., 2009; Wolpert et al., 2011).

**FIGURE 1:**
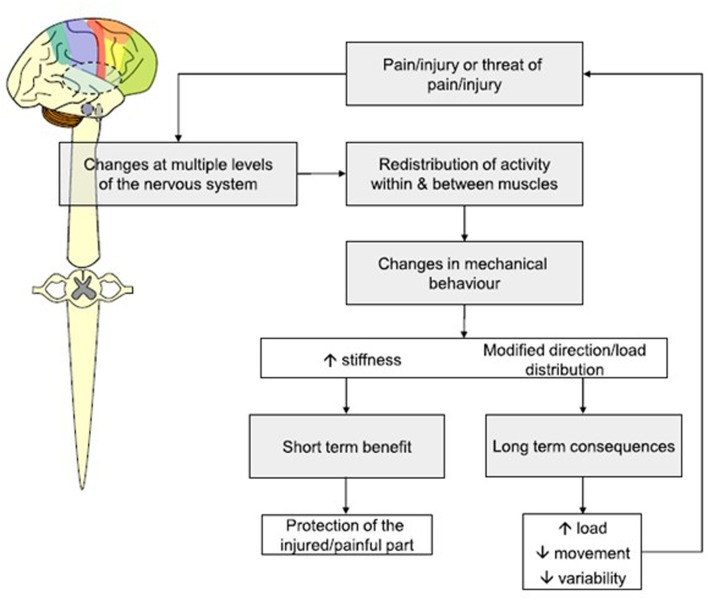
Mechanisms through which pain leads to re-distribution of activity within, and between, muscles. Used by permission from Hodges and Tucker (2011).

Prompted by subtle, but persistent, sensory signals the CNS continually searches for economic trade-offs between desired outcomes, available resources and discomforting perceptions of ‘threat’ (Hodges and Tucker, 2011; Miller et al., 2012). As we accumulate running experiences, we learn to more precisely triangulate between sensory feedback, feedforward activation and desired running outcomes and gravitate toward coordinative solutions more satisfactorily resolving these multiple competing constraints. Progressively, with practice, sensory information and muscular activation strategies co-evolve into a seamlessly integrated sensorimotor system: whereby changes in sensation directly modulate muscular activations, and changes in activation directly modify sensation (Wolpert et al., 2011; see **Figure 1**). Through this elegantly efficient process, sensory feedback information and feedforward activation instructions become irrevocably mutually entangled: preserving running robustness within acceptable limits through an integrated sensorimotor process of ‘self-organizing optimality’ (Glazier and Davids, 2009).

### Organizing Neuro-biological Complexity: Modularity Facilitates Degeneracy

Biological lifeforms are reflectively characterized as complex adaptive systems. Complex: as the behaviors of individual components are inextricably linked to those of multiple others through arrays of processes, cycles and regulatory feedback loops. Adaptive: as the behaviors and collaborative outputs of collections of components flexibly modify their concerted contributions to best fit current context (Manor and Lipsitz, 2013).

Each individual entity within the complex organism is linked, physically or functionally, to every other. Nevertheless there remains an evident modularity, whereby collections of elements are more densely networked to each other than to elements within other modules (Whitacre, 2010; Mason, 2015). All modules are inter-connected, yet are simultaneously partially insulated and functionally semi-autonomous. Modularity, accordingly, facilitates robustness as modules can evolve, reshape, rewire, and repair in tandem, or independently, without jeopardizing survivability of the entire organism (Maleszka et al., 2014; Mason, 2015).

Modularity is a fundamental neuro-biological organizing principle, greatly simplifying otherwise overwhelmingly disordered complexity. Related modules exhibit extensive *functional overlap*, such that alliances of neural networks and peripheral tissues can spontaneously modify behaviors to achieve equivalent ‘outputs’ through a multiplicity of pathways. This functional agility is often conflated with *redundancy*, but is perhaps more reflectively termed *degeneracy* (Glazier and Davids, 2009; Mason, 2015; Seifert et al., 2016). Degeneracy describes the ability of alternate structural pathways to achieve similar functional outcomes in one context, or dissimilar functional outcomes in divergent contexts (Seifert et al., 2016). Degenerate systems are composed of diverse elements, capable of alternately fulfilling similar or overlapping functions and are fundamental facilitators of complexity, robustness, and evolvability (Whitacre, 2010; Maleszka et al., 2014; Mason, 2015). Redundancies, in contrast, occur when sub-sets of identical elements combine to achieve similar outcomes and are subsequently rare, as there are few identical neural and/or biological entities. Degeneracy describes a more flexibly adaptive phenomenon, whereby collaborating communities of fundamentally distinct components produce reliably consistent outputs under fluctuating conditions (Mason, 2015; Seifert et al., 2016).

The human runner represents a highly degenerate system. Consider the phenomenon of leg stiffness during ground-contact—the accurate calibration of which facilitates the protective dampening and economic re-cycling of impact shocks. Our highly degenerate neuro-biological design can produce equivalent leg stiffness’s using diverse coordinative strategies: muscle-tendon units (MTU’s) vary individual contributions whilst, collectively, whole-leg power outputs remain consistent; individual MTU’s achieve similar force outputs by summating different muscle and tendon contributions; individual muscles vary activated motor unit populations under differing contractile conditions to produce identical tensions; multiple combinations of torso, leg and foot postural orientations and pre-set tensionings deliver equivalent propulsive and stabilization-enabling contributions (Wickham and Brown, 1998; Roberts and Azizi, 2011; Turvey and Fonseca, 2014). This option-rich, highly degenerate movement landscape provides a multiplicity of avenues through which collaborating modular alliances combine, and re-combine, to flexibly satisfy dynamically shifting demands.

This degenerate design offers multiple means to accomplish running objectives. Historically, the apparently overwhelming complexity presented by this proliferation of movement ‘options’ was famously interpreted as a control ‘problem’ (Bernstein, 1967). This potentially complex problem, however, is reduced by the gradual construction of synergies—coordinative structures comprised of highly context-specific, context-sensitive functional linkages serving to temporarily constrain collaborating elements such that they act as single coherent units (Latash et al., 2007; Wu and Latash, 2014). Through the formation of synergies the control ‘problem’ is greatly simplified, while simultaneously retaining the benefits of complexity and degeneracy. As such, more recently, the apparent problem of excessive choice has been reframed as the ‘bliss’ of motor abundance (Latash, 2012). When running, this abundance of potentially over-whelming movement ‘choice’ can be, through effective coordination, productively deployed to disperse the running work-burden among networks of collaborating tissues: thereby promoting efficiency and robustness.

### Fractal Variation: Deploying Coordinative Abundance

Conventionally, we equate skilful running with metronomic regularity. As proficient runners achieve reliably consistent stride outcomes, it seems sensible to assume experts precisely replicate running stride characteristics. In recent years, however, close scrutiny of running behaviors illustrates that, even when experts run at steady paces, movement parameters persistently vary (Stergiou and Decker, 2011). Through the lens of traditional motor control paradigms such variability was initially interpreted as ‘noise’—meaningless error arising from the intricacies of the engineering challenge, measurement inaccuracies and fallible biological components. Intriguingly, however, more recent investigations reveal the structure of gait variability to be neither randomly erratic, nor independent of prior events. Instead, the architecture of past, current and future stride variability’s appear statistically linked through, as yet incompletely understood, long-range correlations (Hausdorff, 2007; Stergiou and Decker, 2011; Hamill et al., 2012).

#### Structured Non-random Variability

Mandelbrot’s classic work, *The Fractal Geometry of Nature* (Mandelbrot, 1982), first popularized the term ‘fractal’ to describe the phenomenon, pervasive in Nature, of recurrent structural self-similarity. The unifying characteristic of fractals is scale-free structural replication: whereby individual entities are composed of sub-units of a shared structure, while themselves forming super-ordinate entities conforming to a similarly patterned design. Examples include the branching networks of the vascular system and convoluted folding surfaces of the neo-cortex: both fractally replicating architectures exponentially increasing tissue surface area.

Fractal self-similarity is not, however, confined to physical architectures and also manifests as time-series or organizational replications. Thus sub-regions may be exact or distorted copies of the all-encompassing over-arching structure, or may simply share quantitative, qualitative, or statistical properties (Goldberger et al., 2002; Newell et al., 2005; West, 2010; Vázquez et al., 2016). Fractal signatures are ubiquitous in neurophysiology, with multiple phenomena exhibiting self-similarity across observational scales. Famously, the time series of inter-heartbeat intervals—heart-rate variability—is a fractal phenomenon. Although each beat is unique, its uniqueness is not random but shaped by an innate, neurally embedded background algorithm blending the organism’s unique idiosyncrasies with past experiences, current status, and transient momentary demands, to collectively shape the time-series architecture of the emergent heartbeat (Goldberger et al., 2002). Accordingly the beat-to-beat ‘solution’ to the circulation ‘problem’ is neither tightly prescribed, nor loosely erratic.

Expert running coordination is similarly characterized by the tuned inter-play between predictability and responsiveness bestowed by the fractally fluctuating deployment of option-rich, functionally overlapping degenerate networks. Together, these networks provide the diverse repertoire of behavioral responses essential for survival in chaotic, unpredictable environments (Van Orden, 2007; Nakayama et al., 2010; Stergiou and Decker, 2011; Vázquez et al., 2016).

## Running Variability: Sharing the Running Work-Burden

**FIGURE 2:**
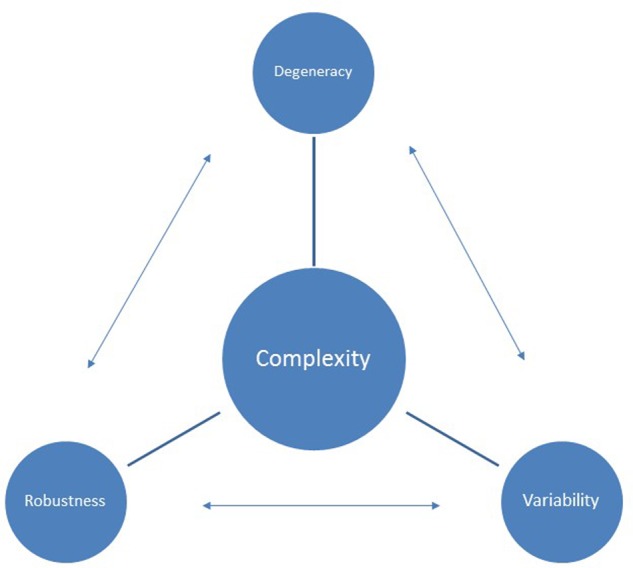
Inter-relationships between complexity and injury resilience.

As with other neuro-biological processes running dynamics exhibit robust fractal characteristics: suggesting stride-to-stride variability is neither random, nor dictated by the fluctuating idiosyncrasies of current conditions. Instead on-going stride variations are meaningfully related—in a decaying Power law fashion—to past variations stretching back over thousands of strides (Meardon et al., 2011; Hamill et al., 2012). This pervasive fractal variation ensures the mechanical stress of running is distributed in ever varying, yet non-randomly organized, patterns: patterns tuned, through practice, to the runner’s unique architectural and experiential peculiarities. This structured variability enables the well-trained runner to disperse the running ‘work burden’ amongst expanded networks of biological tissues, whilst simultaneously retaining the agility to spontaneously respond to emerging challenge (**Figure 2**). Healthy running, accordingly, is characterized by an optimal bandwidth of movement variability: neither too much, nor too little (Meardon et al., 2011; Hamill et al., 2012).

Accordingly proficient running coordination is not the capacity to monotonously replicate an idealized stride pattern, but the ability to continuously recombine expansive, yet conditioned, populations of collaborating neural and biological components. Thereby, enabling the achievement of reliably consistent running outcomes through a diversity of subtly shifting movement permutations.

### Diminishing Complexity, Drives Dysfunctional Variability

As we move through a running life, accumulative cycles of *‘wear and tear’*—of injury, overuse, misuse, and disuse—gradually degrade both the material integrity of biological components and the networked richness of neural connectivity (Elbert and Rockstroh, 2004; Taubert et al., 2010; Hoppeler et al., 2011). As neuro-biological complexity contracts, the landscape of viable degenerate permutations, capable of satisfying running demands, deteriorates. Now, the mechanical stress of running must be distributed amongst shrinking networks of collaborating components (Pelletier et al., 2015).

**FIGURE 3:**
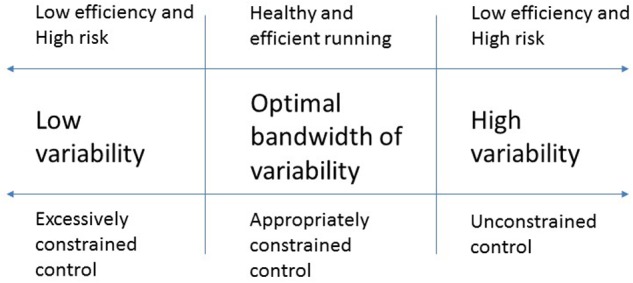
Relationships between running gait variability, risk and efficiency.

Reductions in viable degeneracies do not, however, inevitably decrease running variability. Instead, as the neuro-biological systems struggles to proficiently manage imposed loadings, mechanical stress becomes either more tightly focused on restricted populations of working tissues, or is erratically dispersed amongst expanded webs of unconditioned tissues (Van Orden, 2007; Hamill et al., 2012). As illustration, ACL-deficient knees typically exhibit reduced, whereas ACL-reconstructed knees exhibit dramatically expanded, inter-stride variability (Stergiou and Decker, 2011; Hamill et al., 2012). Such deviations from habituated variability ranges, oscillating between overly formulaic constancy and wild randomness, signify an impaired capacity to absorb, disperse, and purposefully recycle and re-direct impact momentums (Nakayama et al., 2010; **Figure 3**). As coordinative fluency deteriorates, vulnerability to Overuse syndromes and unexpected perturbations escalates.

### Global Accommodation of Local Perturbation

The entangled nature of complex neurobiology ensures that when variability changes at any discrete location, accommodating compensatory behaviors occur elsewhere in the system. As illustration: active injuries typically reduce habitual running variability in the injured leg—constraining control to protect sensitized tissues—, while simultaneously inducing expansions of variability in the non-injured leg (Hamill et al., 2012). Such evidence illustrates that, although running injury is a site-specific event, the accommodation of injury is a system-wide phenomenon occasioning system-wide coordinative adjustments. Importantly, these behavioral modifications, although temporarily functional, inevitably expose compensating tissues to unhabituated loadings. What has not been discussed within the running-related literature, however, are the neural and biological mechanisms which structurally embed running habits and which must be micro-architecturally altered to support coordinative change.

## Pervasive Bio-Plasticity: The Embedded Legacy of Prior Events

A fundamental dimension of human neuro-biology is life-long *experience-dependent plasticity*: the capacity within the CNS and tissues of the periphery to lastingly respond—structurally, chemically, electrically and materially—to repeat experience (Elbert and Rockstroh, 2004; Taubert et al., 2010). Throughout supra-spinal and spinal branches of the CNS persistent patterns of neural activation induce experience-dependent plastic re-configurations, micro-architecturally embedding relationships between regularly co-operating neural components and associated motor units. Plasticity in the CNS is mirrored in the periphery, as tissues re-model in response to habitual loading patterns (Hoppeler et al., 2011). Experience-dependent plasticity refines and economizes communications linkages between collaborating neural networks, and conditions peripheral tissue structures to better cope with regularly encountered movement contexts (Pelletier et al., 2015).

As we converge on individually unique running styles, pervasive neuro-biological plasticity embeds movement habits: thereby constraining the landscape of degenerate movement options to manageable proportions and increasing the probability previously successful ‘solutions’ will be recycled in the future. Plasticity, accordingly, drives the physical embodiment of coordinative change: thereby sculpting the micro-architectural basis of coordinative synergies, linkages, and attractors. Inevitably, however, plasticity is both blessing and curse, and the engraining of new habits inevitably degrades old habits.

### The Plasticity of Over-Specialization

As running experience accumulates, the sensorimotor apparatus becomes ever-more efficient at executing the running task. But neural resources are evolutionarily precious and fundamentally limited commodities and, as such, are persistently re-deployed to fulfill varying roles within diverse tasks. Such conflicting usage patterns drive *competitive plasticity* processes, as neural networks strive to persistently re-model neurological *‘form’* to best fit currently prioritized *‘function.’*

Consequently, as we progress from novice to ‘skilful,’ a by-product of on-going neuroplastic refinement is that fewer networked collaborators are required to manage the evermore highly practiced running pattern (Coq and Barbe, 2011; Avanzino et al., 2014; Pelletier et al., 2015). Subsequently it becomes evolutionarily wasteful to continually dedicate expanded sensorimotor networks to task execution. Accordingly, when the range of running behaviors to which we are regularly exposed becomes monotonously stereotypical, evolutionary pressure to economize resource uptake ensures the landscape of conditioned neural and biological collaborators, dedicated to executing highly practiced running patterns, progressively diminishes as under-utilized resources are re-allocated elsewhere (Elbert and Rockstroh, 2004; Avanzino et al., 2014). A drawback, therefore, of engaging in only a narrow band of overly stereotypical running tasks, is that we become hyper-efficient at deploying reduced populations of degeneracies to execute a narrowing band of self-similar running patterns. As direct consequence, we become increasingly vulnerable to both overuse syndromes, and unhabituated challenges.

### The Plasticity of Disuse

Prolonged abstinence from running drives a progressive loss of physiological conditioning, and also dims the regular flow of running-related sensorimotor information. Consequently, the cortical circuitry normally maintained by consistently processing running-related sensorimotor information is eroded as voracious *competitive plasticity* re-models neural connectivity to best fit current usage patterns (Elbert and Rockstroh, 2004; Pelletier et al., 2015). Subsequently when we return to regular running coordinative control is slightly less proficient and slightly less resilient.

### The Plasticity of Misuse

When we run in injured or irritated states we subtly alter coordination patterns to divert discomforting mechanical stress away from sensitized tissues: alleviating negative sensation, tempering structural damage, and facilitating healing. If, however, we continue to run in compromised patterns for prolonged periods, newly adapted remedial strategies become progressively more plastically engrained within CNS and tissue architectures (Engineer et al., 2012; Avanzino et al., 2014).

The dynamic inter-play between *experience-driven* and *competitive* plasticity processes ensure the traces of temporarily functional coordinative compensations typically remain plastically embedded within neuro-biological structures: thereby becoming the new ‘normal,’ and exerting a legacy not easily erased within the abbreviated timeframes offered by conventional rehabilitation paradigms (Pelletier et al., 2015).

### Promoting Positive Plasticity

Ultimately plastic re-modeling, as it consumes precious material and energetic resources, is evolutionarily expensive (Merzenich et al., 2014; Clark et al., 2015). Within the adult-brain it is not evolutionarily economical to plastically adapt to all stimulation—valuable neural reserves would be immediately depleted. Accordingly, structures in the mature cortex plastically remodel only when specific criteria—regulated by modulatory neurotransmitters such as acetylcholine, dopamine, serotonin, and norepinephrine—are satisfied (Merzenich et al., 2014; Clark et al., 2015). Operating collectively these neuro-modulatory enablers act as “on–off” switches, engaging excitatory and inhibitory processes and temporarily opening plasticity-enabling ‘windows of opportunity’ within which sensorimotor inputs contributing to ‘success’ are selectively amplified; while signals from competing inputs, uncorrelated with that success, are selectively dampened (Merzenich et al., 2014).

Over time, the continued amplification of relevant sensorimotor inputs provides a competitive advantage; greatly enhancing the representational detail embedded in the cortical territory dedicated to processing running-related sensorimotor information (Wolpert et al., 2011; Engineer et al., 2012). Crucially, a core finding emanating from this research domain is that repetitively non-varying, non-challenging ‘mindless’ movements—those not demanding focused attention for satisfactory execution—are insufficiently stimulating to reliably release the cocktail of neuromodulating chemical catalysts necessary for plastic re-modeling within the mature motor cortex (Merzenich et al., 2014; Clark et al., 2015). In contrast, positive plastic re-modeling is optimized in response to behaviorally relevant intense practice, executed at the limits of current abilities and therefore demanding high attentional and motivational drives (Avanzino et al., 2014; Merzenich et al., 2014). Thus positive neural re-modeling is promoted only when tasks are neither so easy that they fail to stimulate focused attention, nor so difficult that continuous failure undermines motivation. In essence coordination improves through engaging challenge, not mindless routine. *A rationale perhaps explaining why rehabilitation processes employing non-challenging coordinative tasks typically fail to generate optimal recovery* (Elbert and Rockstroh, 2004; Merzenich et al., 2014; Clark et al., 2015).

## Conclusion

As we accumulate running experiences, sensory feedback biases us toward personalized styles more satisfactorily resolving achievement of the running objective against an acceptable investment of survival-relevant resources. Guided by innate evolutionary influences, individualized coordinative habits progressively shape around our unique anatomical, biological, neurological, and experiential idiosyncrasies.

Subsequently, as we progress from “novice” to “skilled” runners we more sensitively and smoothly respond to small perturbations, thereby offsetting the need to periodically and clumsily respond to larger challenges as minor errors accumulate. We adjust activation patterns to navigate away from discomforting sensation, thereby moderating tissue aggravations. We gravitate toward activations more proficiently poising bio-composite tissue structures to productively absorb and re-cycle impact momentums, thereby reducing energetic investment and dampening shock decelerations. We learn to exploit our layered landscape of degenerate movement options by fractally varying stride parameters under the integrated influence of historical events and current context, thereby dispersing running work-burdens amongst expanded webs of conditioned tissues. As we accumulate running experience plasticity-processes progressively embed working relationships between regularly collaborating neural components, and embed the tissue features most adaptive to running-specific loadings. As such plasticity is the mechanism that engrains synergies and linkages, and embeds the attractor states underpinning running coordination habits. A key observation, accordingly, is that running coordination change is supported on a platform of neuro-biological plastic modification.

The evolutionary neuro-economics that embed efficient habits, however, eventually encase us within limiting constraints. Plasticity facilitates learning by engraining efficient habits, yet also retains the residues of past traumas and prolonged sensitivities: subsequently ensuring injuries are rarely transient peripheral events, but long-lasting insults etched into cortical tissues of the CNS (Elbert and Rockstroh, 2004; Coq and Barbe, 2011; Pelletier et al., 2015). Similarly the enduring traces of repeated cycles of over-specialization, disuse, and misuse, impose plastic re-configurations not automatically reverting to original conditions once discomfort diminishes and pain-free running is resumed.

As we progress through our running lives, the sensorimotor landscape is in perpetual plastic flux as the integrated influences of health, training, and injury subtly re-configure neural connectivity and biological tissue architectures. Chronically, the progressive accumulation of plastic mal-adaptations drives the creeping decay of networked neural connectivity: compromising sensorimotor information flow, blurring cortical representations of peripheral structures, prompting mal-adaptations in neuronal excitability, and driving disorder within the primary motor cortex (Coq and Barbe, 2011; Avanzino et al., 2014). As a consequence, coordinative control degrades.

When other lifestyle and training considerations—background psycho-emotional stress, monotonous running volumes, generalized and localized fatigue—, are overlaid on already compromised operating conditions, access to expansive populations of viable movement degeneracies further diminishes. As this self-perpetuating cycle escalates, coordinative proficiency decays, susceptibility to tissue irritations grows and we become increasingly fragile to Overuse syndromes and non-formulaic perturbations.

### Practical Insights and Relevance

Deeper appreciation of the various phenomena underpinning running coordination potentially informs many aspects of conventional theory and practice. The topics below are offered as tentative examples:

#### Overuse Injury

Documented incidence rates suggest running-related Overuse injury is neither a ‘solved,’ nor perhaps clearly articulated, problem. Contextualizing Overuse as a direct consequence of chronically compromised coordination emphasizes the necessity of balancing the monotonous stagnation, often implicit in conventional endurance running programs, with the unhabituated challenging stimulation essential to promoting positive neuro-plastic re-modeling. Furthermore, this rationale suggests that introducing coordinative diversity into high-volume running programs may be an effective prophylactic against Overuse occurrence.

#### Enforcing Technical Change

A deeper appreciation of the embedded undercurrents that shape running coordination also questions the long-standing practice of attempting to change technique simply by instructing the runner to consciously re-configure established coordination patterns so as to better conform to an aesthetic ideal. Suddenly altering engrained running habits diverts mechanical stress along unhabituated pathways: thereby inevitably exposing unconditioned tissue to unaccustomed loadings and elevating injury risk. And although empirical evidence remains scarce, there is a suggestion of rising injury rates following short-term technical interventions (see Tucker, 2007).

#### Driving Neuro-plastic Change

Crucially, the perspective presented here suggests we should perhaps pay less attention to how running styles look, and more attention to designing interventions that provide the coordinative challenge necessary to sufficiently stimulate the neuro-plastic re-modeling necessary to persistently refine communicative clarity between CNS and the peripheral musculature. Although such interventions typically fall outside the scope of conventional run-training dogma, many coaches, past and present, have intuitively designed training practices fulfilling the criteria for optimally stimulating neuro-modulatory processes (see for example: Kiely, 2013; Pfaff, personal communication; Smith, personal communication). What emerging scientific insight does add, however, is a growing appreciation of the value of regularly challenging running coordination through the design and implementation of appropriately constructed practices.

#### Visual Evaluation of Running Technique

Conventionally, we associate running coordination with running technique—the visual evaluation of running style evaluated against an aesthetic ideal. This pervasive assumption, however, has never been satisfactorily demonstrated, and no empirical evidence supports a direct relationship between looking ‘better,’ and actually being ‘better.’

When we visually assess a runner’s technique, and extrapolate these observations to running efficiency and injury risk conclusions, we make judgments based on very superficial information. Typically we fail to acknowledge the unseen underlying terrain—the idiosyncratic neurology; the embedded fractal signatures; the unique anatomical architectures and tissue structures; the plastically personalized legacy of historical habits and traumas—upon which coordinative habits are founded. And while it is feasible that, to the highly practiced eye, visual evaluation may provide clues, generally how these clues are interpreted is rooted in assumptions currently lacking an evidence base. Certainly, visual assessments of running proficiency seem unavoidably subjectively biased and previous investigations demonstrate differences in technical ratings between coaches, and even when the same coach evaluates the same footage at different times (Norris et al., 2014).

Would performances improve if running form more closely conformed to perceived technical ideals? Are more aesthetically pleasing runners less injury prone; more economical? While opinions are plentiful, evidence is scarce. *Anecdotally, renowned coach and physiologist, Dr. Jack Daniels, once sent video of 20 physiologically evaluated competitive runners to a selection of coaches and exercise scientists, asking them to—on the basis of visual inspection—rank athletes in order of running economy but, “they couldn’t tell, no way at all” (Kolata, 2007).*

### Finally…

The perspective offered within this review is that coordination is the overarching super-capacity ultimately orchestrating how proficiently neural, muscular, cardiovascular, and metabolic reserves are purposefully harnessed, or wastefully squandered. In relation to running: coordination is the learned deployment of available neuro-biological resources to satisfactorily realize running objectives for an acceptable ‘cost’—in terms of depletion of energetic and neural reserves, and exposure to risk. It is the physical expression of a confluence of psychological, emotional, neural, and biological constraints emerging in response to the on-going interplay between intention, motivation, and perception of risk; informed by emerging sensory feedback; modulated by prior experiences and expectations; biased toward repeatedly re-employing plastically embedded coordinative solutions to current running ‘problems.’

Ultimately, running performance is underpinned by a conglomeration of assorted capacities—cardiovascular, neurological, psychological, physiological, anatomical, muscular, and biomechanical. Yet it is the super-capacity of coordination that regulates how proficiently these overlapping performance contributors are collaboratively expressed to generate propulsive power, promote efficiency, preserve robustness, and accomplish running objectives for an acceptable exposure to discomfort and risk. A deeper appreciation of the underpinnings of the running coordination phenomenon will hopefully enable practitioners to more judiciously design interventions to promote, nurture, and preserve coordinative proficiency in the face of the inevitably accumulating *‘wear and tear’* endured over the course of a running lifetime.

## Author Contributions

The author confirms being the sole contributor of this work and approved it for publication.

## Conflict of Interest Statement

The author declares that the research was conducted in the absence of any commercial or financial relationships that could be construed as a potential conflict of interest.

## References

[B1] Avanzino L. Pelosin E. Abbruzzese G. Bassolino M. Pozzo T. Bove M. (2014). Shaping motor cortex plasticity through proprioception. *Cereb. Cortex* 24 2807–2814. 10.1093/cercor/bht139 23709641

[B2] Bernstein N. A. (1967). *The Co-Ordination and Regulation of Movements.* Oxford: Pergamon Press.

[B3] Bramble D. M. Lieberman D. E. (2004). Endurance running and the evolution of Homo. *Nature* 432 345–352. 10.1038/nature03052 15549097

[B4] Clark D. Schumann F. Mostofsky S. H. (2015). Mindful movement and skilled attention. *Front. Hum. Neurosci.* 9:297 10.3389/fnhum.2015.00297 PMC4484342 26190986

[B5] Clarsen B. Myklebust G. Bahr R. (2013). Development and validation of a new method for the registration of overuse injuries in sports injury epidemiology: the Oslo Sports Trauma Research Centre (OSTRC) overuse injury questionnaire. *Br. J. Sports Med.* 47 495–502. 10.1136/bjsports-2012-091524 23038786

[B6] Coq J. O. Barbe M. F. (2011). “Peripheral and central changes combined induce movement disorders on the basis of disuse or overuse,” in *Movement Disorders: Causes, Diagnoses and Treatments*, ed. Larsen B. J. (Hauppauge, NY: Nova Science Publishers Inc), 2–14.

[B7] Davids K. Glazier P. (2010). Deconstructing neurobiological coordination: the role of the biomechanics-motor control nexus. *Exerc. Sport Sci. Rev.* 38 86–90. 10.1097/JES.0b013e3181d4968b 20335740

[B8] Diedrichsen J. Shadmehr R. Ivry R. B. (2010). The coordination of movement: optimal feedback control, and beyond. *Trends Cogn. Sci.* 14 31–39. 10.1016/j.tics.2009.11.004 20005767 PMC4350769

[B9] Elbert T. Rockstroh B. (2004). Reorganization of human cerebral cortex: the range of changes following use and injury. *Neuroscientist* 10 129–141. 10.1177/1073858403262111 15070487

[B10] Engineer N. D. Engineer C. T. Reed A. C. Pandya P. K. Jakkamsetti V. Moucha R. (2012). Inverted-U function relating cortical plasticity and task difficulty. *Neuroscience* 205 81–90. 10.1016/j.neuroscience.2011.12.056 22249158 PMC3299820

[B11] Gerlach K. E. White S. C. Burton H. W. Dorn J. M. Leddy J. J. Horvath P. J. (2005). Kinetic changes with fatigue and relationship to injury in female runners. *Med. Sci. Sports Exerc.* 37 657–663. 10.1249/01.MSS.0000158994.29358.71 15809566

[B12] Glazier P. S. Davids K. (2009). Constraints on the complete optimization of human motion. *Sports Med.* 39 15–28. 10.2165/00007256-200939010-00002 19093693

[B13] Goldberger A. L. Amaral L. A. Hausdorff J. M. Ivanov P. C. Peng C. K. Stanley H. E. (2002). Fractal dynamics in physiology: alterations with disease and aging. *Proc. Natl. Acad. Sci. U.S.A.* 99(Suppl. 1), 2466–2472. 10.1073/pnas.012579499 11875196 PMC128562

[B14] Hamill J. Palmer C. Van Emmerik R. E. (2012). Coordinative variability and overuse injury. *BMC Sports Sci. Med. Rehabil.* 4:45 10.1186/1758-2555-4-45 PMC3536567 23186012

[B15] Hausdorff J. M. (2007). Gait dynamics, fractals and falls: finding meaning in the stride-to-stride fluctuations of human walking. *Hum. Mov. Sci.* 26 555–589. 10.1016/j.humov.2007.05.003 17618701 PMC2267927

[B16] Hodges P. W. Tucker K. (2011). Moving differently in pain: a new theory to explain the adaptation to pain. *Pain* 152 S90–S98. 10.1016/j.pain.2010.10.020 21087823

[B17] Hoppeler H. Baum O. Lurman G. Mueller M. (2011). Molecular mechanisms of muscle plasticity with exercise. *Compr. Physiol.* 1 1383–1412. 10.1002/cphy.c100042 23733647

[B18] Ivarsson A. Johnson U. Andersen M. B. Tranaeus U. Stenling A. Lindwall M. (2016). Psychosocial factors and sport injuries: meta-analyses for prediction and prevention. *Sports Med.* 47 353–365. 10.1007/s40279-016-0578-x 27406221

[B19] Kelso J. S. (2012). Multistability and metastability: understanding dynamic coordination in the brain. *Philos. Trans. R. Soc. B Biol. Sci.* 367 906–918. 10.1098/rstb.2011.0351 PMC3282307 22371613

[B20] Kiely J. (2013). *The Running Machine Myth. The Running Times.* Available at: http://www.runnersworld.com/race-training/the-running-machine-myth [accessed September 4 2013].

[B21] Kiely J. Collins D. J. (2016). Uniqueness of human running coordination: the integration of modern and ancient evolutionary innovations. *Front. Psychol.* 7:262 10.3389/fpsyg.2016.00262 PMC4826868 27148098

[B22] Kitano H. (2004). Biological robustness. *Nat. Rev. Genet.* 5 826–837. 10.1038/nrg1471 15520792

[B23] Kolata G. (2007). *Running Efficiency: It’s Good, but How Do You Get It? New York Times.* Available at: http://www.nytimes.com/2007/10/11/fashion/11Best.html?pagewanted=all\&_r=1\& [accessed October 11 2007].

[B24] Latash M. L. (2012). The bliss (not the problem) of motor abundance (not redundancy). *Exp. Brain Res.* 217 1–5. 10.1007/s00221-012-3000-4 22246105 PMC3532046

[B25] Latash M. L. Scholz J. P. Schöner G. (2007). Toward a new theory of motor synergies. *Mot. Control* 11 276–308. 10.1123/mcj.11.3.276 17715460

[B26] Maleszka R. Mason P. H. Barron A. B. (2014). Epigenomics and the concept of degeneracy in biological systems. *Brief. Funct. Genomics* 13 191–202. 10.1093/bfgp/elt050 24335757 PMC4031454

[B27] Mandelbrot B. B. (1982). *The Fractal Geometry of Nature.* New York, NY: Henry Holt and Company.

[B28] Manor B. Lipsitz L. A. (2013). Physiologic complexity and aging: implications for physical function and rehabilitation. *Prog. Neuropsychopharmacol. Biol. Psychiatry* 45 287–293. 10.1016/j.pnpbp.2012.08.020 22985940 PMC3568237

[B29] Marcora S. M. Staiano W. Manning V. (2009). Mental fatigue impairs physical performance in humans. *J. Appl. Physiol.* 106 857–864. 10.1152/japplphysiol.91324.2008 19131473

[B30] Mason P. H. (2015). Degeneracy: demystifying and destigmatizing a core concept in systems biology. *Complexity* 20 12–21. 10.1002/cplx.21534

[B31] Meardon S. A. Hamill J. Derrick T. R. (2011). Running injury and stride time variability over a prolonged run. *Gait Posture* 33 36–40. 10.1016/j.gaitpost.2010.09.020 21036046

[B32] Merzenich M. M. Van Vleet T. M. Nahum M. (2014). Brain plasticity-based therapeutics. *Front. Hum. Neurosci.* 8:385 10.3389/fnhum.2014.00385 PMC4072971 25018719

[B33] Miller R. H. Umberger B. R. Hamill J. Caldwell G. E. (2012). Evaluation of the minimum energy hypothesis and other potential optimality criteria for human running. *Proc. R. Soc. B Biol. Sci.* 279 1498–1505. 10.1098/rspb.2011.2015 PMC3282349 22072601

[B34] Mizrahi J. Verbitsky O. Isakov E. Daily D. (2000). Effect of fatigue on leg kinematics and impact acceleration in long distance running. *Hum. Mov. Sci.* 19 139–151. 10.1016/S0167-9457(00)13-10

[B35] Nagengast A. J. Braun D. A. Wolpert D. M. (2010). Risk-sensitive optimal feedback control accounts for sensorimotor behavior under uncertainty. *PLoS Comput. Biol.* 6:e1000857 10.1371/journal.pcbi.1000857 PMC2904762 20657657

[B36] Nakayama Y. Kudo K. Ohtsuki T. (2010). Variability and fluctuation in running gait cycle of trained runners and non-runners. *Gait Posture* 31 331–335. 10.1016/j.gaitpost.2009.12.003 20056419

[B37] Newell K. M. Liu Y. T. Mayer-Kress G. (2005). Learning in the brain–computer interface: insights about degrees of freedom and degeneracy from a landscape model of motor learning. *Cogn. Process.* 6 37–47. 10.1007/s10339-004-0047-6

[B38] Norris M. Anderson R. Kenny I. C. (2014). Method analysis of accelerometers and gyroscopes in running gait: a systematic review. *Proc. Inst. Mech. Eng. P J. Sports Eng. Technol.* 228 3–15.

[B39] Pelletier R. Higgins J. Bourbonnais D. (2015). Is neuroplasticity in the central nervous system the missing link to our understanding of chronic musculoskeletal disorders? *BMC Musculoskelet. Disord.* 16:25 10.1186/s12891-015-0480-y PMC4331171 25887644

[B40] Proske U. Gandevia S. C. (2012). The proprioceptive senses: their roles in signaling body shape, body position and movement, and muscle force. *Physiol. Rev.* 92 1651–1697. 10.1152/physrev.00048.2011 23073629

[B41] Roberts T. J. Azizi E. (2011). Flexible mechanisms: the diverse roles of biological springs in vertebrate movement. *J. Exp. Biol.* 214 353–361. 10.1242/jeb.038588 21228194 PMC3020146

[B42] Saragiotto B. T. Yamato T. P. Junior L. C. H. Rainbow M. J. Davis I. S. Lopes A. D. (2014). What are the main risk factors for running-related injuries? *Sports Med.* 44 1153–1163. 10.1007/s40279-014-0194-6 24809248

[B43] Seay J. F. Van Emmerik R. E. Hamill J. (2011). Low back pain status affects pelvis-trunk coordination and variability during walking and running. *Clin. Biomech.* 26 572–578. 10.1016/j.clinbiomech.2010.11.012 21536356

[B44] Seifert L. Button C. Davids K. (2013). Key properties of expert movement systems in sport. *Sports Med.* 43 167–178. 10.1007/s40279-012-0011-z 23329604

[B45] Seifert L. Komar J. Araújo D. Davids K. (2016). Neurobiological degeneracy: a key property for functional adaptations of perception and action to constraints. *Neurosci. Biobehav. Rev.* 69 159–165. 10.1016/j.neubiorev.2016.08.006 27506266

[B46] Skoyles J. (2008). Respiratory, postural and spatio-kinetic motor stabilization, internal models, top-down timed motor coordination and expanded cerebello-cerebral circuitry: a review. *Nat. Preced.* 1–65. 10.1038/npre.2008.2092.1

[B47] Smirmaul B. D. P. C. (2012). Sense of effort and other unpleasant sensations during exercise: clarifying concepts and mechanisms. *Br. J. Sports Med.* 46 308–311. 10.1136/bjsm.2010.071407 20584757

[B48] Stergiou N. Decker L. M. (2011). Human movement variability, nonlinear dynamics, and pathology: is there a connection? *Hum. Mov. Sci.* 30 869–888. 10.1016/j.humov.2011.06.002 21802756 PMC3183280

[B49] Taubert M. Draganski B. Anwander A. Müller K. Horstmann A. Villringer A. (2010). Dynamic properties of human brain structure: learning-related changes in cortical areas and associated fiber connections. *J. Neurosci.* 30 11670–11677. 10.1523/JNEUROSCI.2567-10.2010 20810887 PMC6633410

[B50] Todorov E. (2004). Optimality principles in sensorimotor control. *Nat. Neurosci.* 7 907–915. 10.1038/nn1309 15332089 PMC1488877

[B51] Todorov E. (2009). Efficient computation of optimal actions. *Proc. Natl. Acad. Sci. U.S.A.* 106 11478–11483. 10.1073/pnas.0710743106 19574462 PMC2705278

[B52] Tucker R. (2007). *Pose Running Reduces Running Economy: The Missing Study.* Available at: http://sportsscientists.com/2007/10/pose-running-reduces-running-economy/ [accessed October 3 2007].

[B53] Turvey M. T. Fonseca S. T. (2014). The medium of haptic perception: a tensegrity hypothesis. *J. Mot. Behav.* 46 143–187. 10.1080/00222895.2013.798252 24628057

[B54] van der Worp M. P. Ten Haaf D. S. van Cingel R. de Wijer A. Nijhuis-van der Sanden M. W. Staal J. B. (2015). Injuries in runners; a systematic review on risk factors and sex differences. *PLoS ONE* 10:e0114937 10.1371/journal.pone.0114937 PMC4338213 25706955

[B55] Van Orden G. C. (2007). The fractal picture of health and wellbeing. *Psychol. Sci. Agenda* 21 1–5.

[B56] Vázquez P. Hristovski R. Balagué N. (2016). The path to exhaustion: time-variability properties of coordinative variables during continuous exercise. *Front. Physiol.* 7:37 10.3389/fphys.2016.00037 PMC4753307 26913006

[B57] West B. J. (2010). Fractal physiology and the fractional calculus: a perspective. *Front. Physiol.* 1:12 10.3389/fphys.2010.00012 PMC3059975 21423355

[B58] Whitacre J. M. (2010). Degeneracy: a link between evolvability, robustness and complexity in biological systems. *Theor. Biol. Med. Model.* 7:6 10.1186/1742-4682-7-6 PMC2830971 20167097

[B59] Wickham J. B. Brown J. M. M. (1998). Muscles within muscles: the neuromotor control of intra-muscular segments. *Eur. J. Appl. Physiol. Occup. Physiol.* 78 219–225. 10.1007/s004210050410 9720999

[B60] Wolpert D. M. Diedrichsen J. Flanagan J. R. (2011). Principles of sensorimotor learning. *Nat. Rev. Neurosci.* 12 739–751. 10.1038/nrn3112 22033537

[B61] Wu Y. H. Latash M. L. (2014). The effects of practice on coordination. *Exerc. Sport Sci. Rev.* 42 37–42. 10.1249/JES.0000000000000002 24188981 PMC3897239

